# Supplementary material for: Temperature dependence of crystal melt coexistence for supported polyethylene filaments
Source: Nat Commun. 2025 Dec 13;16:11127. doi: 10.1038/s41467-025-67465-2 (PMC12706021; doi:10.1038/s41467-025-67465-2)
Supplement: Supplementary file 1 — Supplementary Information [file 41467_2025_67465_MOESM1_ESM.pdf]

Supplementary Information

for

Temperature dependence of crystal – melt coexistence for supported polyethylene nanofibers

Da Huang (黄达)[1], Thorsten Hugel[2], Bizan N. Balzer[2,3], Günter Reiter[1]✉

[1] Institute of Physics, University of Freiburg, Germany

[2] Institute of Physical Chemistry, University of Freiburg, Germany

[3] Freiburger Materialforschungszentrum (FMF), University of Freiburg, Germany

✉ guenter.reiter@physik.uni-freiburg.de

Content

1: For the definition of W_{soft} **(Page 2)**

2: A set of force spectroscopy measurements taken at successive positions along the cross-section of a filament **(Page 7)**

3: AFM phase images from filaments decorated with "beads-on-a-string" patterns **(Page 12)**

4: AFM phase images showing that W_{soft} is qualitatively the same for cantilevers of different stiffness **(Page 13)**

5: Examples of AFM phase images from various filaments measured at different temperatures **(Page 14)**

6: Converting the units of the oscillating cantilever from the measured voltage (in units of V) into the actual amplitude (in units of nm) **(Page 15)**

7: The size-dependent melting temperature of the PE filaments **(Page 16)**

8: Dependence of the AFM phase signal on the scanning orientation relative to the height gradient of the sample. **(Page 17)**

1. For the definition of W_{soft}

In our experiments, we used the viscoelastic properties of the surrounding mica substrate as a reference. Accordingly, we set the values of the corresponding phase and height data of the surrounding mica substrate to zero. Furthermore, the "soft zone", is defined as the area with a negative phase value. The "hard core" of the filaments is defined as the area with a positive phase value.

We define the value of W_{soft}, which characterizes the width of the soft region at the periphery of the filaments, as the full width at half maximum of the peak observed in the cross-section of the AFM phase signal. This cross-section was taken in the direction normal to the three-phase contact line.

As explained in the main text, the penetration depth d_{p} depends on the intermittent-contact force f_{T} , which, in turn, depends in an approximate linear relation $f_{\text{T}} \sim 1 - s$ on the set point ratio s. Furthermore, d_{p} depends also on the elastic modulus (E) of the sample at position (x, y). Thus, d_{p} is recorded as $d_{\text{p}}(x, y, f_{\text{T}}, E)$.

However, if the thickness $\big(h(x, y)\big)$ of a soft sample at position (x, y) is smaller than d_{p} and the underlying solid substrate is not deformable (has a high elastic modulus), the AFM phase signal will have contributions of both, the soft sample and the hard substrate. The higher f_{T}, the stronger is the contribution of the hard substrate. Thus, for the meniscus-shaped region of the soft layer at the periphery of the filaments, especially when they are small in height and width and in particular at positions (x, y) close to the threephase contact line, the AFM cantilever tip can easily pierce through the whole soft layer if f_{T} is sufficiently large.

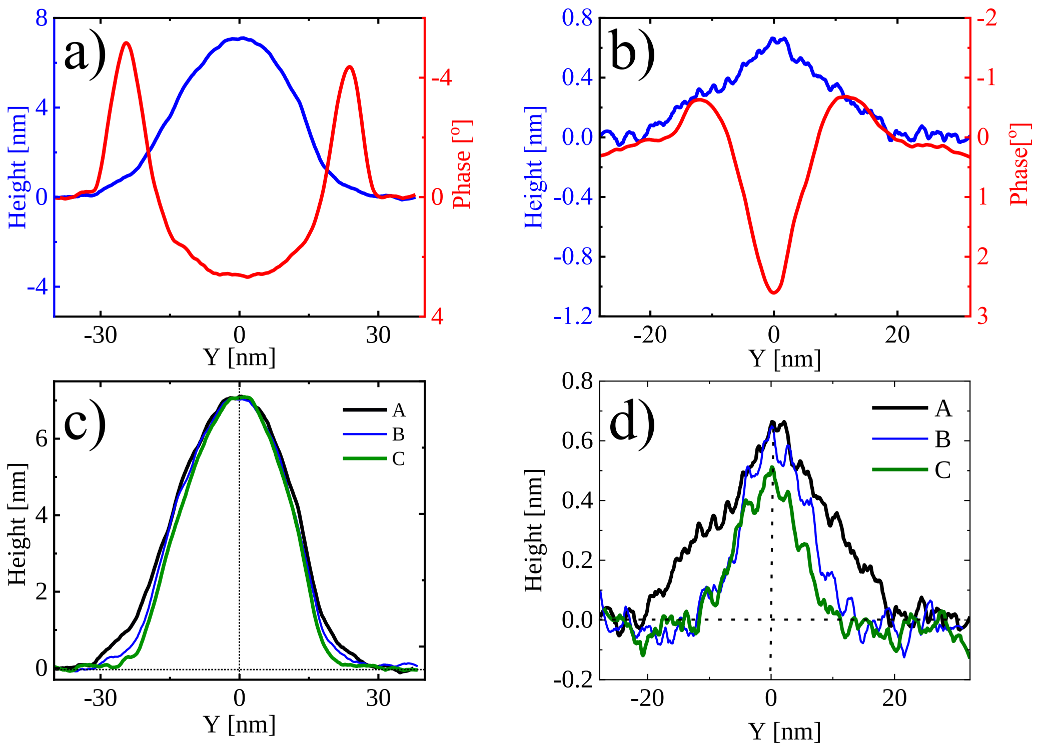

Fig. SI-1: Height $\widehat{H}(x, y, f_T, E)$ and phase cross-section data from (a) a thick and (b) a thin filament, respectively. (a): Results taken from Figure 1 (e) of the main text compared with (b) analogous results from a thinner filament with a height less than approximately 1 nm. (c and d) Corresponding height cross-sections taken at increasing values of the intermittent-contact force f_T. Results shown in (c) were taken from Figure 2 (a) of the main text. For curves A, B and C, the set point ratio s decreased in steps of ca. 0.01, starting from $s = 0.995 \pm 0.005$. The corresponding increase in intermittent-contact force f_T caused a stronger deformation, especially in the soft part at the boundary of the filament.

In our experiments, we have varied the intermittent-contact force f_T in discrete steps of Δf_T proportional to $\Delta s = 0.005$, which is close to the experimental resolution limit of f_T. For the smallest experimentally possible intermittent-contact force proportional to the highest set point ratio of $s = 0.995 \pm 0.005$, it is unclear if the AFM cantilever tip pierces the soft layer completely. From our AFM observations, we concluded that the height (and width) of the filaments affect the thickness of the soft region at the periphery of the filaments. Thus, we have compared our results, shown in Figure 2 of our main text, for varying values of s from a ca. 7 nm high filament, with results obtained from a filament with a height less than approximately 1 nm. This comparison is shown below in Figures SI-1 and SI-2.

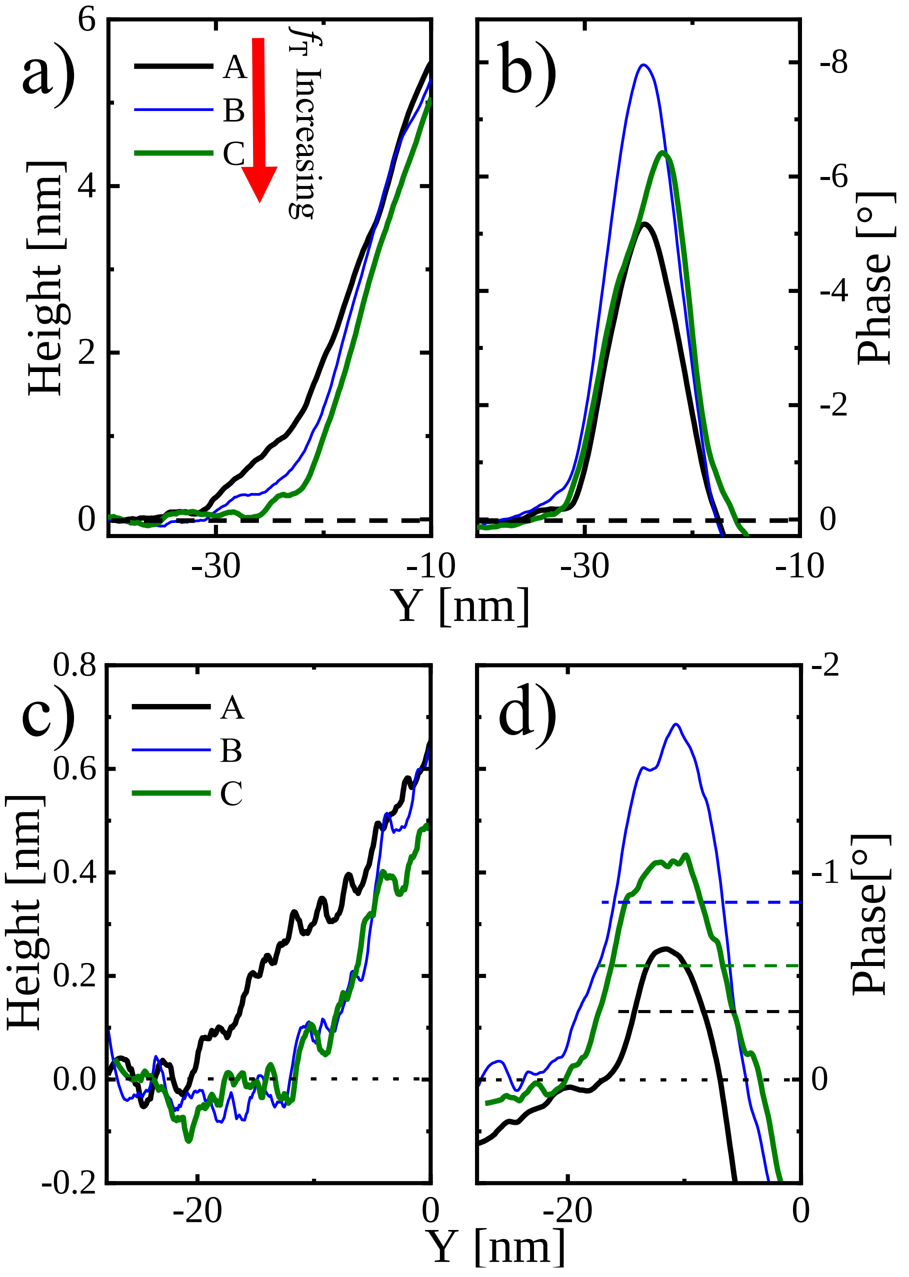

Fig. SI-2: Focusing on the soft region close to the periphery of the filaments shown in Fig.SI-1, we highlight the impact of increasing intermittent-contact force f_T on the corresponding changes in height and phase values. (a) and (b) are extracted from Figures 2(b) and 2(c) of the main text. (c) and (d) show the corresponding zoom-ins of the soft region of the thin filament presented in Fig. SI-1 (d). The set point ratio s decreased in steps of ca. 0.01, starting from $s = 0.995 \pm 0.005$ to $s = 0.975 \pm 0.005$ for A→ C, respectively, corresponding to discrete steps in increasing the intermittent-contact force f_T. The three dotted lines in (d) indicate the half maximum of the peak in the phase signal for data measured for the three different set point ratio s. The full width of the peaks at these positions is taken as our measure for W_{soft}.

As can be seen clearly from Figure SI-2 (d) for the thin filament, the value of the full width at half maximum of the peak in the phase signal, which we use for determining the value of W_{soft}, can depend strongly on the used intermittent-contact force f_{T} and the corresponding penetration depth d_{p}. Ultimately, for high intermittent-contact forces, determination of W_{soft} would also be erroneous as the value of W_{soft} would tend to zero. At such high forces, the AFM cantilever tip pierces the soft region completely and the signal will be almost exclusively determined by the properties of the underlying substrate, i.e., the values of the phase signal and the measured height $\widehat{H}(\mathrm{x}, \mathrm{y}, f_{\mathrm{T}})$ approach the reference values of the substrate (which both have been set to zero) for the whole range of the soft region. In our experiments, we have avoided this limit by using rather weak intermittent-contact forces.

However, for a weak and constant intermittent-contact force, the value of W_{soft}, determined through the full width at half maximum of the peak in the phase signal, may depend also on the thickness of the soft layer. Therefore, we cannot use the same value of the set point ratio for all filaments independent of their size (height and width). Thus, we tried to identify the appropriate value of s for filaments of different heights (characterized through the value of the maximum height (H_{MAX}) of the filament derived from cross-sections taken in the direction perpendicular to the three-phase contact line) for which the value of W_{soft} reached a maximum.

For example, for $s = 0.975 \pm 0.005$ in Figure SI-2 (a), within the soft region (characterized by negative values of the AFM phase signal) we obtained $\widehat{H}(\mathrm{x}, \mathrm{y}, f_{\mathrm{T}}) \approx 0$, signifying that the intermittent-contact force was high enough to pierce through the whole soft region. For the significantly thinner filament investigated in Figure SI-2 (c), $\widehat{H}(\mathrm{x}, \mathrm{y}, f_{\mathrm{T}}) \approx 0$ was observed in the corresponding soft region already for the higher set point ratio $s = 0.985 \pm 0.005$.

Using the criterion of the largest value of W_{soft} obtained for an appropriately chosen value of s (for example the green line in Fig. SI-2), we established the results shown in Figure SI-3 for many filaments differing in their maximum height (H_{MAX}). Within the uncertainty of these measurements, W_{soft} was independent of H_{MAX}.

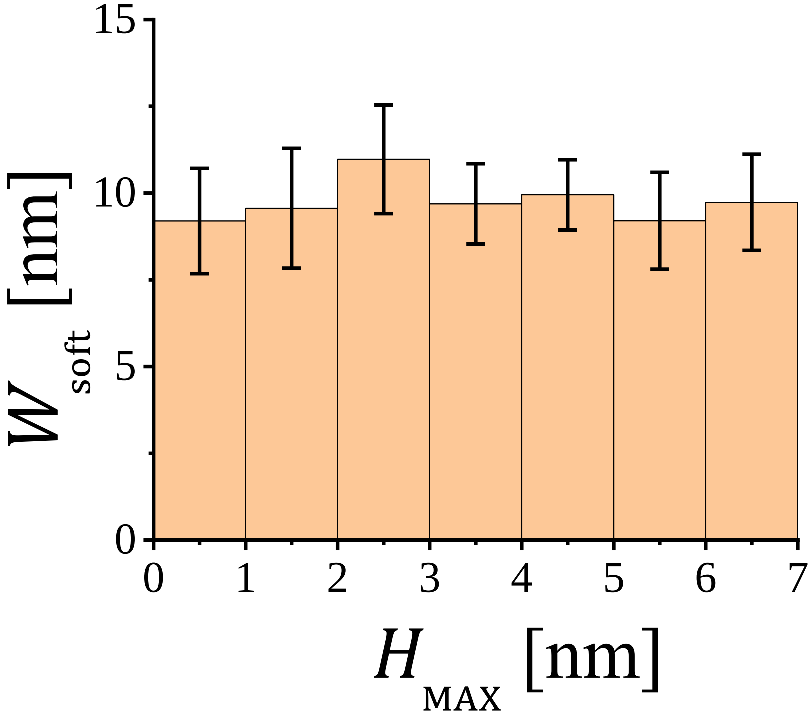

Fig. SI-3: Statistical data showing that W_{soft} is independent of H_{MAX}. The error bars represent the standard deviation. The histogram is derived from 56 individual measurements.

2. A set of force spectroscopy measurements taken at successive positions along the cross-section of a filament

The interpretation of the structure of the investigated PE filaments described in our manuscript is supported by a set of force spectroscopy measurements shown below (Fig.SI-4 a). This set of 10 force-distance curves was taken in small steps of around 3 nanometers at different positions along the cross-section of a PE filament (the measured positions are indicated in Fig.SI-4 b). The force-distance curves 1 and 2 were measured on a mica substrate. The peak in the retraction part (labeled with a red arrow) indicates the point, at which the cantilever tip started to detach the mica surface.[1] When the spectrum was measured on the "soft PE layer", an increase in energy dissipation was expected, which was indeed observed in force-distance curves 3 and 4. One observed an increase of the area between approach and retraction (a hysteresis, which is related to energy dissipation). The force-distance curve at the position of the "solid PE core" of the filament revealed that the cantilever tip penetrated the nanometer-sized solid PE-filament at a high enough force, leading to a decrease in the force during the approach (break-through). That is observed in both force-distance curves 5 and 6, labeled with a black arrow. Force-distance curves 7, 8, and 9 represent the "soft PE layer", similar to force-distance curves 3 and 4, but were measured on the opposite side of the filament. Finally, the series of measurements passed across the whole width of the PE filament and the final force-distance curves 10 was measured again on the mica surface, which is identical to force-distance curves 1 and 2.

For comparison, force-distance curves 1, 7, and 6 from Fig.SI-4 (a) are superposed in Fig.SI-4 (c), representing spectra taken on mica (black), the "soft PE layer" (blue), and the solid PE core (red), respectively. Besides, the velocity of the tip, during both the approach and the retraction, is set to 100 nm/s; the sampling rate is 2048 data points per second; the waiting time, after the setpoint is reached, is 0 seconds.

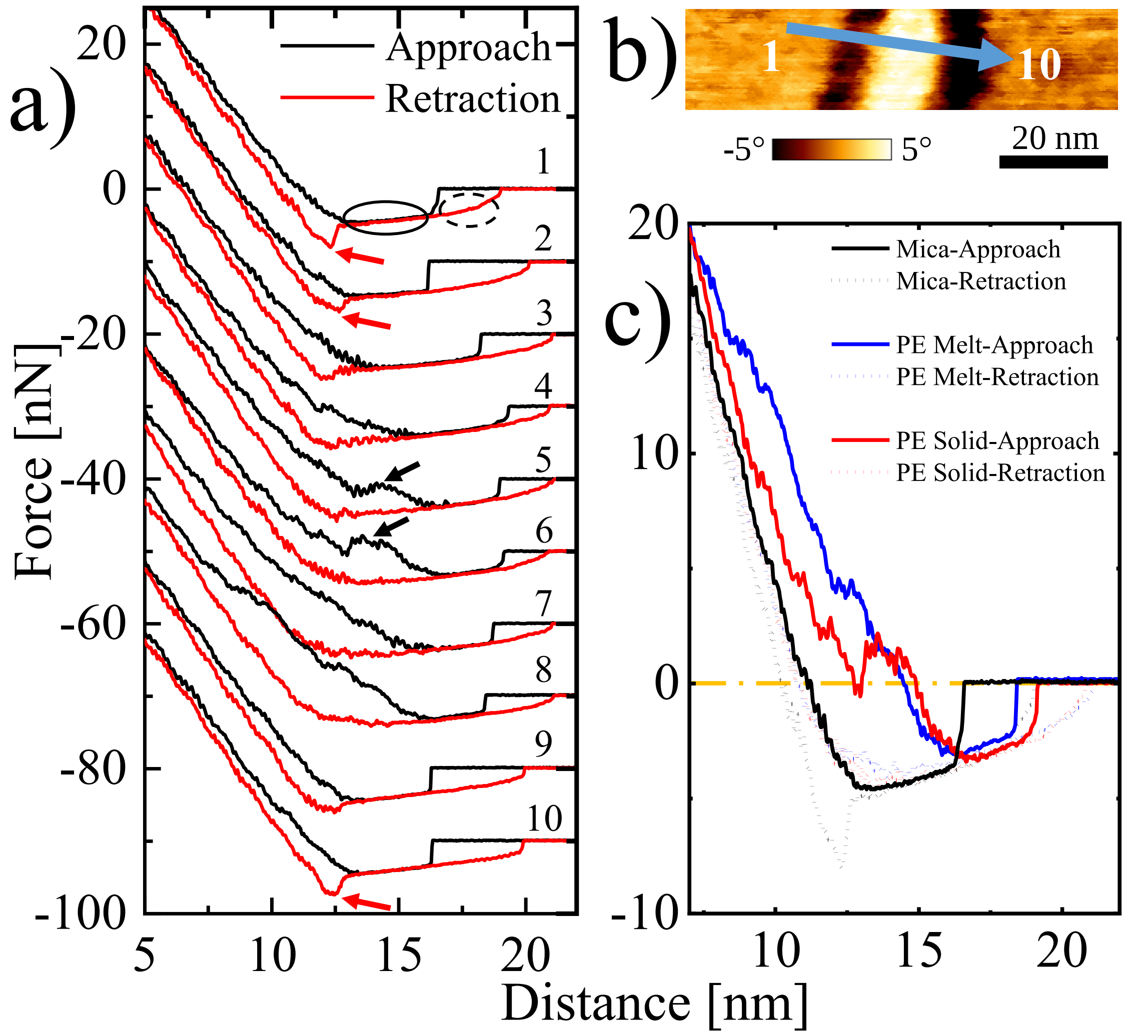

Fig. SI-4: (a) A set of force-distance traces taken with a PPP-NCSTR cantilever across a PE filament on a mica substrate, moving from one side (number 1) to the other side (number 10). The set of traces was taken along the direction indicated by the blue arrow shown in the AM-AFM phase image (b). For comparison, each force-distance curve in (a) was shifted down by 10 nN on the vertical axis (relative to the previous one). The constant base line of curve 1 at large distances was set to zero, the retraction direction was set as positive distance direction. (c) Superposition of force-distance curves 1, 6, and 7 shown in (a), representing the mica substrate (black), the solid region of the PE filament (red), and the melt region of the PE filament (blue), respectively. The yellow dash-dotted line in (c) indicates the position of zero force. The spring constant of the cantilever was 5 N/m (calibrated using the thermal noise method[2]). The position labeled with a red arrow in the retraction curve indicates the position where the cantilever tip detached from the mica surface. The position labeled with a black arrow in the approach curve indicates a breaking of the nanometer-sized solid PE filament (the "shish") due to the force applied by the cantilever tip, thus leading to a decrease in the force upon further indentation of the cantilever tip. After the jump-in contact, there is a region with almost no change in force value (solid ellipse), and the long tail on the retraction curve (dashed ellipse). Those phenomena indicate that the sample surface or the cantilever tip is covered (or contaminated) by liquid.

The experiments, described in detail below, indicate how to find the location of a force-distance trace (measured in contact mode) in its corresponding AM-AFM image. The cantilever "PPP-NCSTR" was selected for the experiment because it was designed for AM-AFM and has relatively low (to “TAP190Al-G” cantilevers with around 45 N/m) spring constant of 5 N/m. Using the same cantilever, we measured images in the contact mode and the AM mode, respectively. First, an AM-AFM image was taken for a selected filament. In the following, after retracting the cantilever tip for a few micrometers, we switched the AFM from the AM mode to the contact mode. Finally, the cantilever tip was brought in contact with the sample surface, and an AFM image was taken in contact mode. By comparing the image taken in the contact mode with the height image of the AM-AFM measurement, the selected filament could be identified and located. As the cantilever tip was only moved a few micrometers in the z-direction, we are confident that the same filament was measured in both cases. Finally, the target positions were selected on the contact mode image according to the AM-AFM phase image that was taken in the first part of the experiment. For these selected target positions, a set of force-distance curves was acquired.

In force spectroscopy measurements conducted on the region of the mica substrate surrounding our UHMWPE filaments — specifically, lines 1, 2, and 10 in Fig. SI-4(a) — a distinctive feature was observed: an area with nearly constant force value following the jump-in to contact (indicated by a solid ellipse) accompanied by a long tail in the retraction curve (indicated by a dashed ellipse). This suggests the presence of a liquid medium between the sample surface and the AFM tip. Possible sources for this liquid medium include a water meniscus formed via Kelvin condensation, a thin layer of molten polyethylene (PE), or contamination of the cantilever tip by molten PE chains.

To identify the nature of this liquid medium, deflection–distance measurements were performed using a new cantilever having a clean AFM tip (no contamination) on a freshly cleaved mica surface. The same cantilever tip was then used to perform measurements on the mica region of the UHMWPE sample. As shown in **Fig. SI-5**, the "nearly-constant force region" (indicated by a solid ellipse) and the "extended tail region" (indicated by a dashed ellipse) were observed on the deflection–distance measurement on a freshly cleaved mica surface with a new cantilever having a clean AFM tip (no contamination). We attribute the apparent anomalies in the curves to the formation of a water meniscus between the AFM tip and the sample. Under ambient conditions, the water meniscus is caused by Kelvin condensation, which occurs universally. Thus, we

observed water menisci also for the PE filaments, providing a consistent explanation for the nearly-constant force region (solid ellipse) observed immediately after the jump-to-contact. Similarly, the extended tail visible during retraction (dashed ellipse) is a characteristic feature of contact with a water meniscus which eventually ruptures.

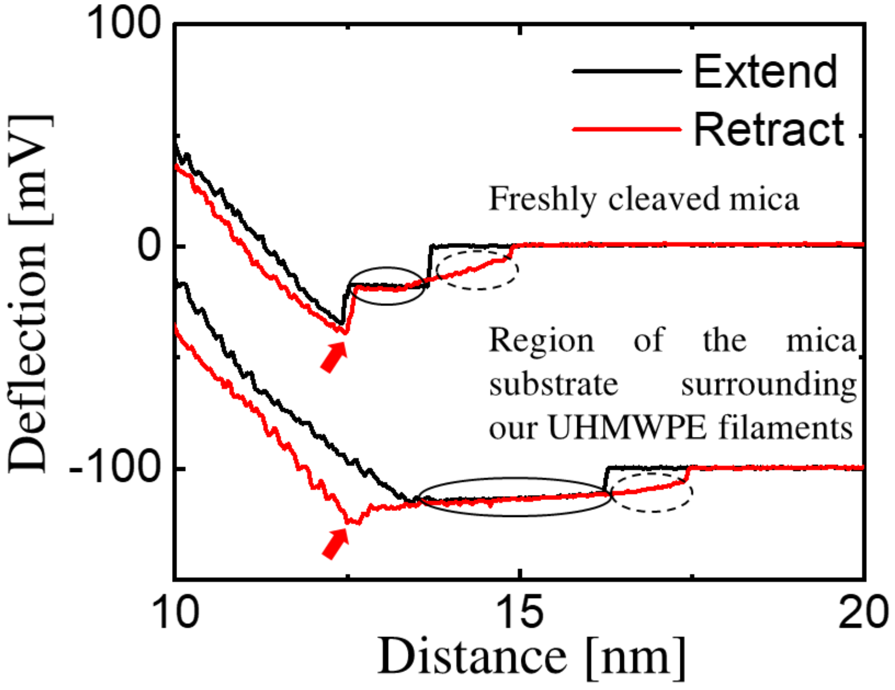

Fig. SI-5: Deflection-distance traces taken with the same PPP-NCSTR cantilever tip. The measurement was first done on a freshly cleaved mica surface using a new AFM cantilever tip (top). Then, with the same cantilever tip, measurement was done on the region of the mica substrate surrounding our UHMWPE filaments (bottom). During both measurements, the surrounding humidity was 34% RH, and the temperature was 22 °C.

Furthermore, a comparison between deflection–distance traces obtained on freshly cleaved mica and on the region of the mica substrate surrounding our UHMWPE filaments revealed a notably narrower region of a nearly-constant force on the pure mica substrate. This difference suggests the presence of an extremely thin, uniform layer of molten UHMWPE covering the mica surface in our samples. The uniformity of this molten UHMWPE layer is demonstrated by the homogeneous value of the AFM phase within the whole region of the mica substrate surrounding our UHMWPE filaments, which was observed consistently throughout our study. The existence of such an ultrathin layer of molten UHMWPE is expected due to the strongly different values of the surface tension of mica and polyethylene, lowering the surface tension of the region of the mica substrate

surrounding our UHMWPE filaments. Additionally, a pronounced jump in the deflection of the cantilever (marked by the red arrows in **Fig. SI-5**) is evident in both approach and retraction traces on fresh mica, but appears only in the retraction direction of the curves measured on the region of the mica substrate surrounding our UHMWPE filaments. This discrepancy occurs because, as the AFM tip approached the surface, the ultrathin layer of molten UHMWPE initially “shields” the tip from strong attractive interactions with the actual (bare) mica surface. However, as this layer was penetrated during further extension, upon subsequent retraction the trace exhibited a sudden detachment event when the tip lost contact with this layer. Moreover, due to the ultrathin layer of molten UHMWPE, this detachment "jump" during retraction is less sharp on the UHMWPE-covered mica than on freshly cleaved mica.

Knowing that both, a water meniscus and an ultra-thin polyethylene (PE) melt layer cover the entire UHMWPE sample, a critical question arises: whether the phase value from the mica substrate surrounding our UHMWPE filaments can still serve as a reliable reference for flattening the AFM phase data, and, if this flattening is necessary

As the absolute phase baseline may vary between scan lines due to factors such as thermal drift or environmental fluctuations, the value of the phase signal representing the mica substrate surrounding our UHMWPE filaments was used as a reference, specifically for flattening the AFM phase images. It is also important to note that taking the region of the mica substrate surrounding our UHMWPE filaments as the reference surface, this region may include additional interfacial layers, such as a thin layer of molten UHMWPE melt, contributions from the water meniscus formed under ambient conditions, or even potential tip contamination from the UHMWPE melt. Nevertheless, as we always used the same referencing procedure under these conditions, our interpretation remains both methodologically consistent and physically justified, in particular as the overall response of the phase signal of this composite reference system remains stable and invariant over time. Crucially, any systematic shift in the reference value does not affect our interpretation of the presented phase contrasts relative to this reference value. The clear distinction in phase values between the filament and the mica substrate surrounding our UHMWPE filaments, as well as the contrast between the two distinct regions within the filament itself, remains unequivocally discernible regardless of baseline adjustments.

3. AFM phase images from filaments decorated with “beads-on-a-string” patterns

As shown in Figure SI-6, within the uncertainty of the measurements and for a given temperature (here, this is room temperature), all values of W_{soft} are the same for all samples, independent of the local morphology of the filaments. The value of W_{soft} is almost constant along the whole filament, including the region around the “beads”.

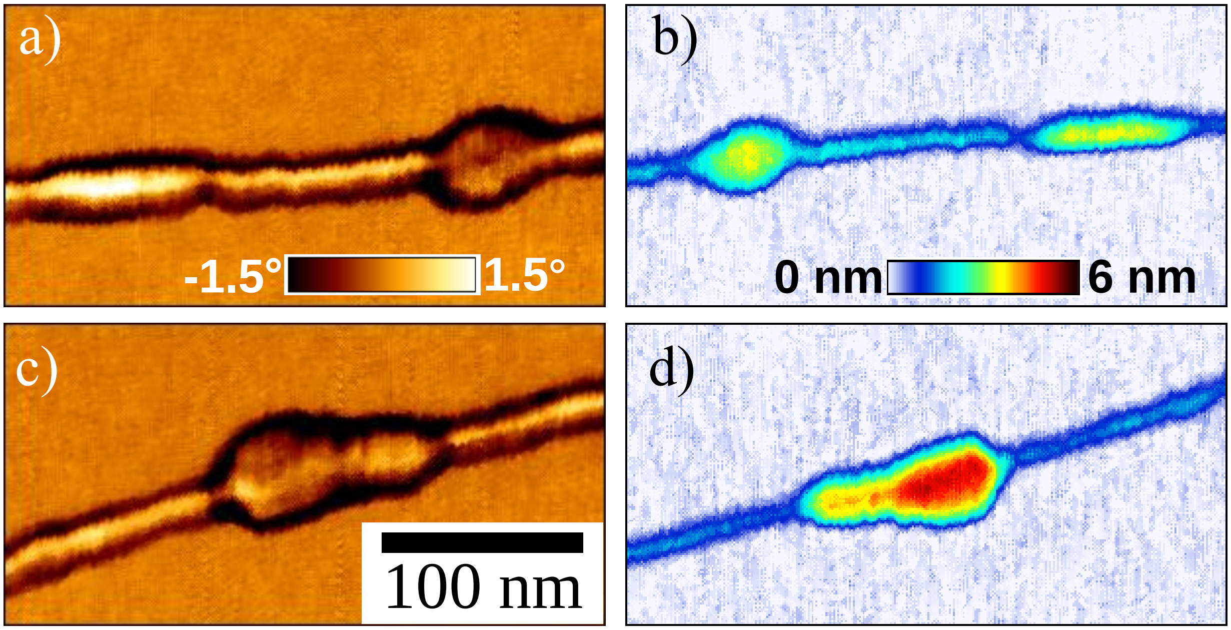

Fig. SI-6: AFM phase (a, c) and corresponding height (b, d) images of filaments decorated with “beads-on-a-string” patterns, from samples prepared with **PE-M** and measured at room temperature with a set point ratio $s = 0.990 \pm 0.005$. The scale bar and the color code apply to both images.

4. AFM phase images showing that W_{soft} is qualitatively the same for cantilevers of different stiffness

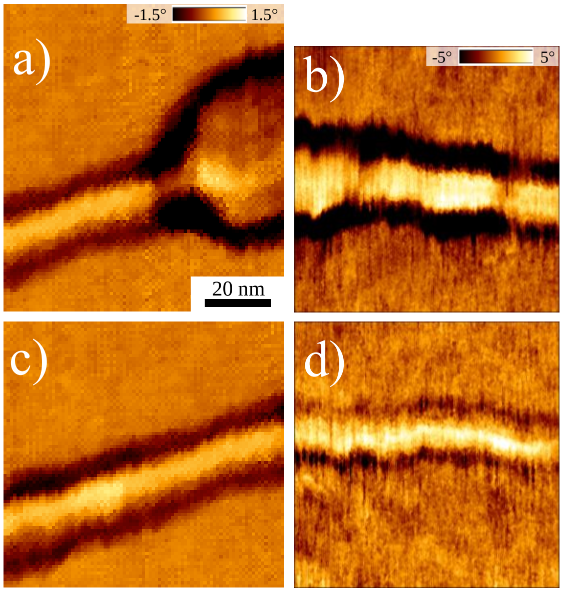

Fig. SI-7: AM-AFM phase images taken with different types of cantilevers: For a and c, we used "TAP190Al-G" cantilevers and for b and d, we used "PPP-NCSTR" cantilevers, respectively. Images c and d were taken on straight filaments while images a and b were taken on beads-on-a-string structures.

In Fg.SI-7, AFM phase images were taken with two different cantilever types. For images a and c, we used "TAP190Al-G" cantilevers, while for images b and d we used "PPP-NCSTR" cantilevers. Images c and d were taken on straight filaments (both of the filament have a height of around 1 nm), while images a and b were taken on beads-on-a-string structures.

We note that the spring constant for the cantilever “TAP190Al-G" was around 45 N/m, while it was around 5 N/m for the cantilever "PPP-NCSTR" (calibrated using the thermal noise method[2]). Both, for “TAP190Al-G" and "PPP-NCSTR", the cantilever tip had a radius of curvature of ca. 10 nm. The color code for images a and c is the same, ranging from -1.5 deg to 1.5 deg. For images b and d, the color code ranges from -5 deg to 5 deg. The setpoint ratio for all images in Fig.SI-7 was fixed to $s = 0.99 \pm 0.005$. The “free” oscillation amplitude A_0 for images a and c was in the range of 72 to 80 nm, while for images b and d, it was in the range of 52 to 57 nm. For all cases shown in Fig.SI-7, the value of W_{soft} is rather uniform along the whole filaments and for the various measurement conditions, consistent with the theoretical interpretation of our observations as described in our manuscript.

5. Examples of AFM phase images from various filaments measured at different temperatures

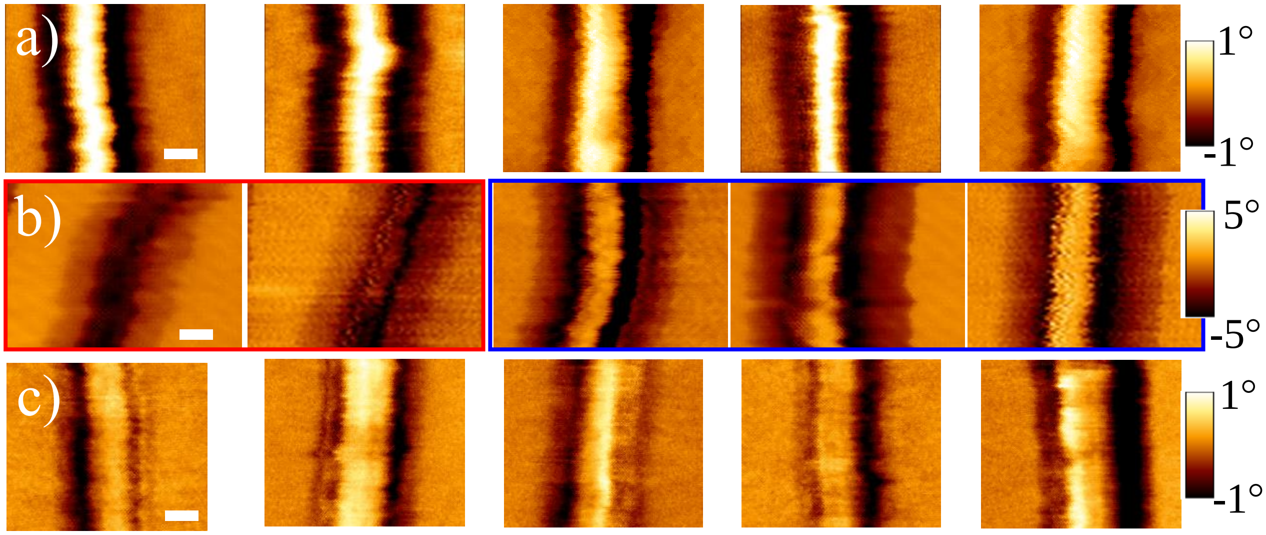

Fig. SI-8: AFM phase images from different filaments on a spin-coated PE-M sample taken at (a) room temperature before annealing, (b) at 100 °C (after heating up and equilibrating the sample for more than 40 min) and (c) at room temperature (after cooling back and equilibrating the sample for more than 40 min). In row (b), the images within the red box are derived from thin filaments with $\boldsymbol{H_{\mathrm{MAX}}} < \mathbf{2\ nm}$. The images within the blue box are taken from thick filaments with $\boldsymbol{H_{\mathrm{MAX}}} >$ $\mathbf{6\ nm}$. The length of the white scale bar is 10 nm and applies to all images in an individual row. All measurements were performed with a set point ratio of $\boldsymbol{s} = \mathbf{0.990 \pm 0.005}$.

Interestingly, for the thin filaments in Figure SI-7(b) – red box, the whole filament became molten at 100 °C, i.e., the value of the AFM phase was negative within the whole filament, while for the thick filaments in Figure SI-7 (b) – blue box, a central hard region (expressed by positive values of the AFM phase) still remained at this elevated temperature of at 100 °C. That means: thinner (narrower) filaments will melt at lower temperatures than wider ones.

Furthermore, the comparison of images in row (a) with images in row (c) shows that the changes in the value of W_{soft} induced by increasing the temperature were fully reversible within the uncertainty of these AFM measurements.

6. Converting the units of the oscillating cantilever from the measured voltage (in units of V) into the actual amplitude (in units of nm)

The amplitude inverse optical level sensitivity (amplitude invOLS), determined by indentation into mica (Muscovite, quality: V1, from Plano GmbH) for the different types of cantilevers (as shown in fig.SI-9) used in this manuscript are: for "TAP190Al-G", from "BudgetSensors", it is (36.5 $\pm$ 0.5) nm/V and for "PPP-NCSTR", from "NANOSENSORS", it is (26 $\pm$ 1) nm/V. The mean values of the amplitude invOLS were derived from 4 independent measurements. For all the AM-AFM images shown in our manuscript, we used cantilevers of the type "TAP190Al-G". There, we applied a "free" amplitude of $A_0 = 2 - 2.2$ V, which thus corresponds to $A_0 \approx 73 - 80$ nm.

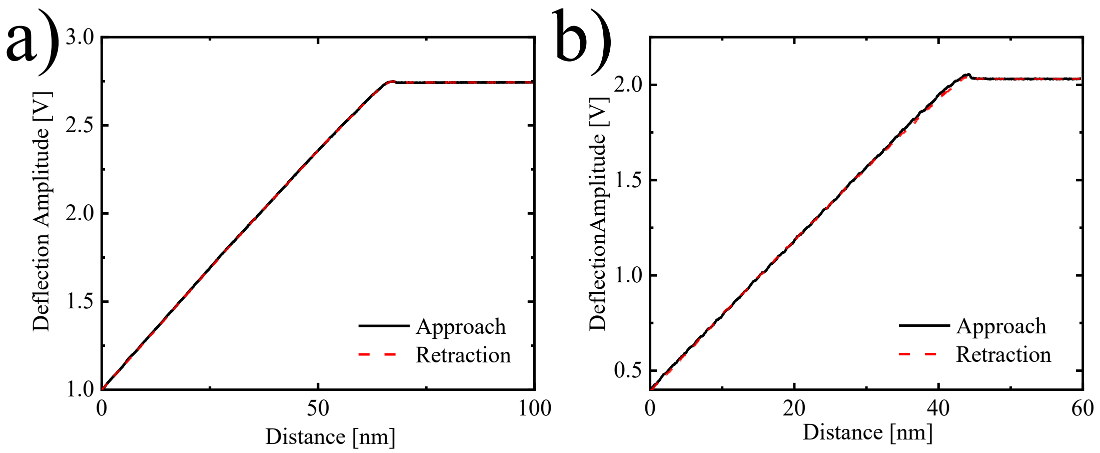

Fig. SI-9: The deflection amplitude (in units of V) as a function of distance moved by the piezo in the z-direction (related to a reduced oscillation amplitude due to the tip-mica interactions, i.e. a hard mica surface was not deformed while the amplitude of the cantilever was limited by the proximity of the hard mica surface) for two different types of cantilevers. (a) "TAP190Al-G" and (b) "PPP-NCSTR". The setpoint value was chosen to obtain a deflection amplitude value of the cantilever of 1 V (for TAP190Al-G) or 0.4 V (for PPP-NCSTR). The direction of retraction is positive. In both images, the black line is plotted with data collected during the approach of the cantilever to the mica surface, and the red dashed line is during the retraction of the same from the mica surface.

We notice that the amplitude invOLS measurements in this section were performed in a **dynamic** mode, **not a static** mode of AFM. During the measurement, a cantilever was driven by applying a constant oscillation force. As the cantilever approached a hard surface, the deflection amplitude as

a function of the relative position of the cantilever decreased once the distance between the cantilever and the surface was approximately equal to the deflection amplitude. Once the cantilever tip was in (intermittent) contact with the underlying mica surface (Muscovite, quality: V1, from Plano), which represents a hard (non-deformable) surface with respect to the cantilever stiffness, the motion of the piezo in the z-direction was causing a linear decrease of the deflection amplitude of the cantilever [3]. From this decrease as a function of the distance moved in the z-direction, the amplitude invOLS was calculated. Besides, the velocity of the tip, during both the approach and the retraction, is set to 100 nm/s; the sampling rate is 2048 data points per second; the waiting time, after the setpoint is reached, is 0 seconds.

7. The size-dependent melting temperature of the PE filaments

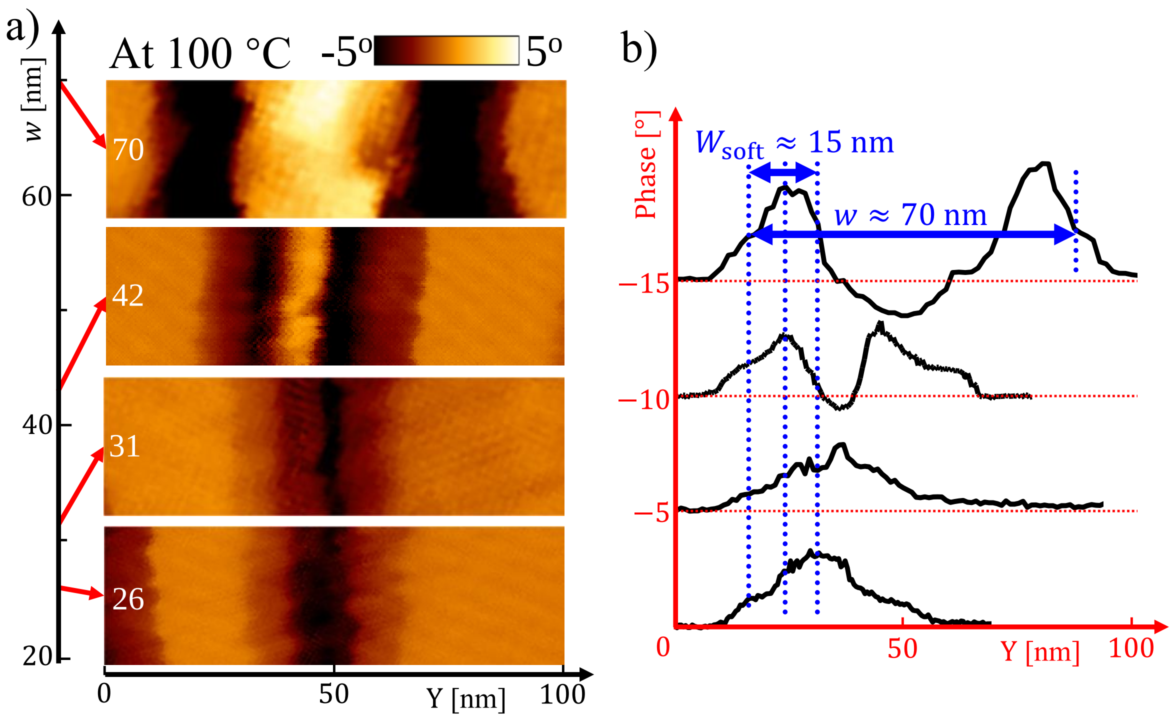

Fig. SI-10: (a) AFM phase images of filaments with different widths (w, is labeled in each image, bottom-to-top = narrow-to-wide) on spin-coated PE-M at 100 °C. (b) Corresponding phase cross-sections, which were vertically shifted by 5° each for better comparison. The uniform soft layer thickness ($W_{soft} \approx 15\ nm$) is consistent with the statistical data presented in Figure 5 of the main text. The filament width (w) is defined as the lateral distance between the half-height points of the two opposing soft layers as schematically illustrated in (b).

Our AFM phase images (Fig. SI-10a) and the corresponding cross-sections (Fig. SI-10b) provide direct experimental evidence for the relationship between filament width and melting behavior. At 100°C, we observe that filaments satisfying the condition $w \lesssim 2 \cdot W_{\mathrm{soft}}$ (where $W_{\mathrm{soft}} \approx 15$ nm yielding $w \approx 30$ nm) are completely molten. In contrast, wider filaments ($w > 2 \cdot W_{\mathrm{soft}}$) consistently show a hard crystalline core bounded by molten layers. The width of the crystalline core increases systematically with the width of the filament. These measurements quantitatively confirm our hypothesis: For $2W_{\mathrm{soft}}(T_{\mathrm{exp}}) \approx w \rightarrow T_{\mathrm{m}}(w) = T$. These results highlight the relationship between (1) surface premelting effects, (2) the critical size required for secondary nuclei for polymer crystal growth, and (3) melting point depression.

8. Dependence of the AFM phase signal on the scanning orientation relative to the height gradient of the sample.

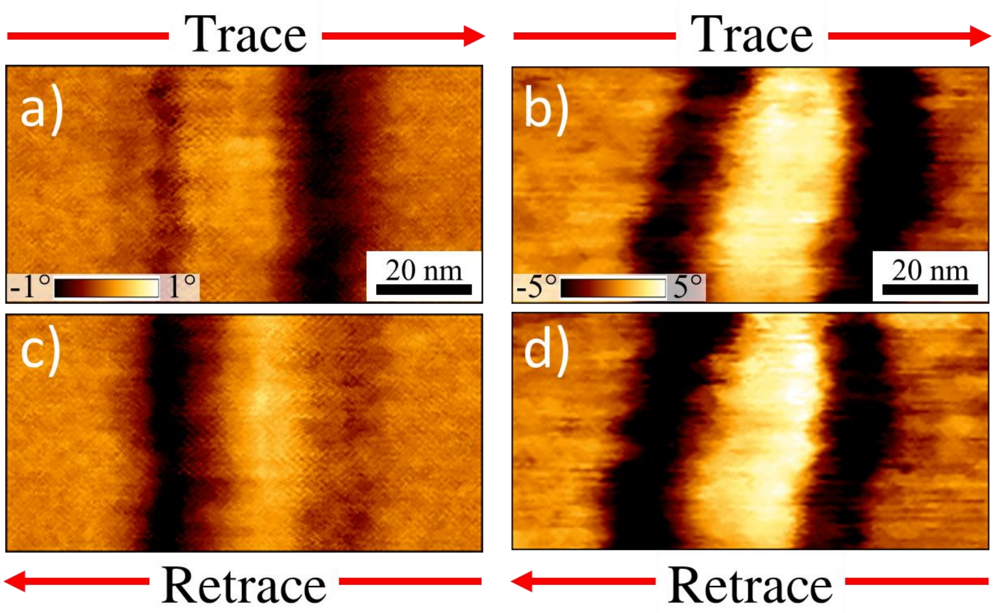

Fig. SI-11: AFM phase trace and corresponding retrace images indicate that the observed asymmetry is a result of whether the AFM tip is scanning in trace or retrace direction. Trace (a) and retrace (c) AFM phase images taken from the same filament (taken in a single AFM measurement under ambient conditions), similar to the condition of Fig. SI-8. Both images have the same color code and scale bar. Trace (b) and retrace (d) AFM phase images taken from the same filament (taken in one AFM measurement under ambient conditions), similar to the condition of Fig. SI-4. Both images have the same color code and scale bar. The scan direction of those images is labeled on their top (bottom) with a red arrow.

The asymmetry observed in the soft layers in Supplementary Figs. SI-4 and SI-8, particularly when scanning perpendicular to the filament axis, are likely influenced by the scanning orientation relative to the height gradient of the sample.

In **Fig. SI-11**, we have included both trace and retrace phase images for two representative filaments. The observed asymmetries are mainly related to the direction of scanning over the sample topography. Specifically, when scanning perpendicular to the filament, the tip encounters an ascending and then descending slope, resulting in phase profiles in the trace and retrace directions that exhibit "mirror-image asymmetry" — as clearly demonstrated in **Fig. SI-11**.

To minimize potential errors introduced by this effect, all values of W_{soft} reported in the manuscript were measured from cross-sectional phase profiles obtained when the cantilever was scanning in the direction of **ascending** the soft layer slope. For instance, as illustrated in **Fig. SI-11**, the width of the left soft layer was measured from the trace scan (where the tip ascends the left slope), while the right soft layer was measured from the retrace scan (where the tip ascends the right slope). This approach ensured consistent and reliable quantification of the soft layer width, reducing artifacts related to scan direction.

References:

(1) Weisenhorn, A. L.; Hansma, P. K.; Albrecht, T. R.; Quate, C. F. Forces in Atomic Force Microscopy in Air and Water. *Applied Physics Letters* **1989**, *54* (26), 2651–2653. https://doi.org/10.1063/1.101024.

(2) Hutter, J. L.; Bechhoefer, J. Calibration of Atomic‐force Microscope Tips. *Review of Scientific Instruments* **1993**, *64* (7), 1868–1873. https://doi.org/10.1063/1.1143970.

(3) Anczykowski, B.; Krüger, D.; Fuchs, H. Cantilever Dynamics in Quasinoncontact Force Microscopy: Spectroscopic Aspects. *Phys. Rev. B* **1996**, *53* (23), 15485–15488. https://doi.org/10.1103/PhysRevB.53.15485.
